# The efficacy of cognitive behavior stress management on functional dyspepsia: A protocol for systematic review and meta-analysis

**DOI:** 10.1097/MD.0000000000029157

**Published:** 2022-05-20

**Authors:** Min Cheng, Xiu-E. Zhou, Yu-Chen Xu, Hong-Mei Dou

**Affiliations:** a Digestive Endoscopy Center, Taizhou People's Hospital, Jiangsu, China; b Department of Gastroenterology, Taizhou People's Hospital, Jiangsu, China; c Department of Radiology, Taizhou People's Hospital, Jiangsu, China; d Department of Operation Room, Taizhou People's Hospital, Jiangsu, China.

**Keywords:** cognitive behavior stress management, functional dyspepsia, meta-analysis, systematic review

## Abstract

**Background:**

Functional dyspepsia and digestive disorders are common, debilitating and costly. Little information is available about the role of stress management in terms of cognitive-behavioral treatment of dyspepsia. We performed a protocol for systematic review and meta-analysis to evaluate the effectiveness of cognitive behavior stress management for the treatment of functional dyspepsia.

**Methods:**

A comprehensive search of several databases from 1966 to March 2022 will be conducted. The databases include Ovid Medline In-Process & Other Non-Indexed Citations, Ovid MEDLINE, Ovid EMBASE, Ovid PsycINFO, Ovid Cochrane Central Register of Controlled Trials, Ovid Cochrane Database of Systematic Reviews, and Scopus. The primary outcome for this study was the rate of successful treatment (presence of no more than mild pain or discomfort after treatment). The secondary outcomes were improvement of dyspepsia at short-term (<1 year) and long-term (≥1 year) follow up, improvement in quality of life, and development of treatment-related adverse events. The risk of bias in each included study will be assessed utilizing the Cochrane Collaboration's risk of bias tool. The Review Manager 5.3 (Cochrane Collaboration, Oxford, UK) will be used to analyze the data.

**Results:**

We will synthesize the current studies to evaluate the effectiveness and safety of cognitive behavior stress management on functional dyspepsia.

**Conclusion:**

The result of this review will provide more reliable references to help clinicians make decisions when dealing with functional dyspepsia.

## 1 Introduction

Functional gastrointestinal disorders are common unexplained gastrointestinal symptom complexes that are thought to arise from different regions of the gastrointestinal tract, and the two most recognized disorders are functional dyspepsia and the irritable bowel syndrome. Functional dyspepsia is a clinical syndrome comprising chronic symptoms arising from the gastroduodenal region.^[1,2]^ According to the Rome criteria, based on expert consensus, the prototypical symptoms are bothersome recurrent postprandial fullness, inability to finish a normal sized meal (early satiety), epigastric pain or epigastric burning in the setting of a normal upper endoscopy.^[3,4]^ However, many patients with functional dyspepsia also experience other troublesome symptoms including nausea, bloating, belching, and heartburn.^[5,6]^

Although the course of functional dyspepsia is not associated with mortality, ultimately an important issue is that this disease typically affects the lives of patients and the associated economic costs of care in the community.^[7,8]^ On the other hand, functional dyspepsia is a kind of psychosomatic disorder and some researchers have confirmed stable somatization in these patients. Somatization functional gastrointestinal disorders are accompanied with psychiatric disorders. Thus, considering the role of psychological and social factors and the accompanied symptoms of psychological disorders in patients with functional dyspepsia, researchers have become interested in the use of complementary therapies, that is, alternative and psychological.

Cognitive behavior stress management refers to a family of stress management therapy which focuses on cognitive-behavioral approach.^[9]^ Elements of cognitive-behavior therapy include cognitive restructuring, raising awareness about stress, relaxation training, problem-solving training, self-management, and adequate social support. Stress is an important factor affecting the symptoms of patients with functional dyspepsia^[10]^; on the other hand, stress management increases the ability of people to reduce stress and appropriate compatibility with stressful situations.^[11]^ Currently, little information is available about the role of stress management in terms of cognitive behavioral treatment of functional dyspepsia. Thus, we performed a protocol for systematic review and meta-analysis to evaluate the effectiveness of cognitive behavior stress management for the treatment of functional dyspepsia.

## 2 Material and methods

This protocol is reported following the preferred reporting items for systematic reviews and meta-analysis protocols (PRISMA-P) statement guidelines.^[12]^ We have registered this study at Open Science Framework (OSF, https://osf.io/). The registration DOI of this study is 10.17605/OSF.IO/RPWK8. Ethical approval is not required for this study since it relies on secondary data.

### 2.1 Inclusion criteria

#### 2.1.1 Study type

In this work, we will include randomized controlled trials (RCTs) of cognitive behavior stress management on functional dyspepsia in adult populations (≥18 years). Non-RCTs and observational study will be excluded. Studies published in English and Chinese will be included.

#### 2.1.2 Types of patients

This study will include patients diagnosed with functional dyspepsia after biochemical, endoscopy, and ultrasound reviews. Included patients had no restrictions on age, sex, economic status, severity of the disease, or education.

#### 2.1.3 Intervention type

The control group received placebo or conventional pharmacotherapies recommended by guidelines, and the intervention group received additional cognitive-behavioral stress management.

#### 2.1.4 Outcomes

The primary outcome for this study was the rate of successful treatment (presence of no more than mild pain or discomfort after treatment). The secondary outcomes were improvement of dyspepsia at short-term (<1 year) and long-term (≥1 year) follow up, improvement in quality of life, and development of treatment-related adverse events.

### 2.2 Search methods

A comprehensive search of several databases from 1966 to March 2022 will be conducted. The database includes Ovid Medline In-Process & Other Non-Indexed Citations, Ovid MEDLINE, Ovid EMBASE, Ovid PsycINFO, Ovid Cochrane Central Register of Controlled Trials, Ovid Cochrane Database of Systematic Reviews, and Scopus. Two authors will independently draft and carry out the search strategy. In addition, we manually retrieve other resources, including the reference lists of identified publications, conference articles, and gray literature. The key terms used for the search are “functional dyspepsia,” “cognitive behavior stress management,” and “randomized controlled trial”.

### 2.3 Data extraction

We will extract and record the first author's name, year of publication, study design, group information, age, gender, dropouts, sample size, duration of intervention, outcomes, and adverse effects from the studies that met the inclusion criteria. We will contact the corresponding authors for additional information if necessary.

### 2.4 Risk of bias assessment

The risk of bias in each included study will be assessed utilizing the Cochrane Collaboration's risk of bias tool.^[13]^ Two researchers will independently evaluate the bias based on the following items: random sequence generation, allocation concealment, blinding of the participants and personnel, blinding of the outcome assessments, incomplete outcome data, selective reporting, and other sources of bias. The studies will be evaluated as low risk, high risk, and unclear risk. Inconsistencies will be resolved by discussion with other reviewers.

### 2.5 Data analysis

The Review Manager 5.3 (Cochrane Collaboration, Oxford, UK) will be used to analyze the data. For outcomes, we will use relative risk and 95% confidence interval to evaluate dichotomous outcomes, while using standardized mean difference with 95% confidence interval to assess continuous variables. The heterogeneity between RCTs will be calculated by Cochrane *χ*^2^ and *I*^2^ tests. If *P* ≥ .05 and *I*^2^ ≤ 50%, no statistical heterogeneity is observed, the data will be calculated with a fixed-effect model. If *P* < .05 and *I*^2^ > 50%, the random effect model will be used. If there is significant heterogeneity, subgroup analysis will be conducted based on different interventions, controls, durations of treatment, and outcome measures. We will carry out sensitivity analyses to investigate the robustness of the study conclusions. In this way, we will be able to assess the impact of low-quality studies on the overall results and whether the results are robust.

### 2.6 Assessment of publication biases

A funnel plot analysis will be drawn to assess the publication bias and Egger test in Stata 14.0 (Stata Corp, College Station, TX) will be conducted for statistical investigation.

### 2.7 Assessment of quality of evidence

We will use the Grading of Recommendations Assessment, Development, and Evaluation^[14]^ to assess the results. In the Grading of Recommendations Assessment, Development, and Evaluation system, the quality of evidence will be categorized into 4 levels: high, moderate, low, and very low quality.

## 3 Discussion

Functional dyspepsia is common and recent studies indicated a heterogeneity of this disorder whose specific clinical symptom patterns could be related to varied gastric pathophysiological mechanisms.^[15]^ Psychological factors have also been associated with functional dyspepsia.^[16,17]^ Clinical observations suggested a higher anxiety level and stress experienced in functional dyspepsia patients with a positive correlation to the disease severity.^[18,19]^ Cognitive-behavioral stress management has been applied successfully in many physical and emotional conditions. It includes 2 goals:

(1) to accept the existence of stress events, as well as its harm to health, and
(2) to provide effective methods to relieve stress. But it has not yet been fully applied to functional dyspepsia. Therefore, there is a need to clarify and illustrate the function and action modes.

To the best of our knowledge, this is the first meta-analysis of RCTs to evaluate the effectiveness of cognitive behavior stress management for the treatment of functional dyspepsia. We hope that the result of this review will provide more reliable references to help clinicians make decisions when dealing with functional dyspepsia.

## Author contributions

**Data analysis:** Xiu-E. Zhou.

**Data collection:** Yu-Chen Xu.

**Study design:** Hong-Mei Dou.

**Writing:** Min Cheng.
